# Neurite Growth and Polarization on Vitronectin Substrate after in Vitro Trauma is not Enhanced after IGF Treatment

**DOI:** 10.3390/brainsci8080151

**Published:** 2018-08-11

**Authors:** K. Bergen, M. Frödin, C. von Gertten, A. -C. Sandberg-Nordqvist, M. K. Sköld

**Affiliations:** 1 Department of Neuroscience, Karolinska Institutet, 171 77 Stockholm, Sweden; karin.bergen@ki.se (K.B.); magnus.frodin@ki.se (M.F.); 2 Department of Clinical Neuroscience, Karolinska Institutet, 171 76 Stockholm, Sweden; Christina.Von.Gertten@ki.se (C.v.G.); anki.sandberg.nordqvist@ki.se (A.-C.S.-N.); 3 Neurosurgery Unit, Department of Neuroscience, Uppsala University, 751 85 Uppsala, Sweden

**Keywords:** IGF-1, IGFBP-2, vitronectin, in vitro trauma, regeneration, hippocampal neuron

## Abstract

Following traumatic brain injuries (TBI), insulin-like growth factor (IGF) is cortically widely upregulated. This upregulation has a potential role in the recovery of neuronal tissue, plasticity, and neurotrophic activity, though the molecular mechanisms involved in IGF regulation and the exact role of IGF after TBI remain unclear. Vitronectin (VN), an extracellular matrix (ECM) molecule, has recently been shown to be of importance for IGF-mediated cellular growth and migration. Since VN is downregulated after TBI, we hypothesized that insufficient VN levels after TBI impairs the potential beneficial activity of IGF. To test if vitronectin and IGF-1/IGFBP-2 could contribute to neurite growth, we cultured hippocampal neurons on ± vitronectin-coated coverslips and them treated with ± IGF-1/IGF binding protein 2 (IGFBP-2). Under same conditions, cell cultures were also subjected to in vitro trauma to investigate differences in the posttraumatic regenerative capacity with ± vitronectin-coated coverslips and with ± IGF-1/IGFBP-2 treatment. In both the control and trauma situations, hippocampal neurons showed a stronger growth pattern on vitronectin than on the control substrate. Surprisingly, the addition of IGF-1/IGFBP-2 showed a decrease in neurite growth. Since neurite growth was measured as the number of neurites per area, we hypothesized that IGF-1/IGFBP-2 contributes to the polarization of neurons and thus induced a less dense neurite network after IGF-1/IGFBP-2 treatment. This hypothesis could not be confirmed and we therefore conclude that vitronectin has a positive effect on neurite growth in vitro both under normal conditions and after trauma, but that addition of IGF-1/IGFBP-2 does not have a positive additive effect.

## 1. Introduction

The insulin-like growth factor system (IGFs) is one example of growth factors that show widespread cortical upregulation following traumatic brain injury (TBI) [1,2]. This family of growth factors is developmentally regulated and displayed in specific regions of the brain, which suggests explicit physiological roles [3]. Increased levels of mRNA coding for these molecules are observed during development, following trauma, and in certain brain tumors [4,5]. Following CNS injury, important roles for IGF regarding the recovery of neuronal tissue, plasticity, and neurotrophic activity are implicated. IGF-1 promotes the survival of cerebellar neurons through the inhibition of apoptosis induced by low intracellular potassium. Therapeutic benefits associated with IGF-1 treatment of brain injury are suggested to derive from both its positive effects on neuronal survival and its inhibition of the glial inflammatory reaction, via the regulation of NF-κB [6]. The gene expression of both IGF-1 and IGF-2 is increased in the cerebral cortex after hypoxic-ischemic injury [7,8]. Cytotoxic colchicine lesions of the dentate gyrus have been shown to increase IGF-1 and IGFBP-2 mRNA levels in microglia [9] and intraventricular treatment with IGF linked to hippocampal neurogenesis [10,11]. We have shown that IGF-1, IGFBP-2, and IGFBP-4 are strongly upregulated following cerebral cortical contusion [1]. The increase in IGF-1 mRNA is predominantly found at the contusion site, while the increase in IGFBP-2 and IGFBP-4 mRNA is more widespread in the ipsilateral cortex with neuronal localization [1].

It has also been shown that the injection of IGF-1 leads to the rescue of neurons following hypoxic-ischemic injury and TBI [12]. Displacement of endogenous IGFs from IGFBPs by blocking binding sites using IGFBP ligand inhibitors seems to be a potential treatment after injury [13]. A clinical trial, phase II study, has shown that pharmacological concentrations of IGF-1 may improve outcomes in patients with moderate-to-severe head injury [14]. Åberg et al. showed that the peripheral infusion of IGF-1 selectively induces neurogenesis in the adult rat hippocampus [15]. Evidence that IGF-1 rescues motor neurons has led to therapeutic trials of human recombinant IGF-1 in ALS patients [16]. However, the results were not satisfying, which might be explained by results from Wilczak, showing that ALS patients have an increased amount of IGFBPs. The strong binding capacity for IGFBPs to IGF-1 leads to reduced levels of free bioavailable IGF-1 [17]. However, to a high degree, it is still unclear if and how the IGF system can play a key role in posttraumatic neural survival and regeneration.

Neuronal polarization, i.e., the specialization of neurites into axons and dendrites, is a critical step in the process of neuronal maturation and development. Neuronal polarity is essential for the correct flow of electrical signals between communicating neurons and is thus a requirement for the functional integrity of the nervous system [18]. The first step in neuronal polarization is the establishment of an axonal identity in one of the neurites, which occurs during stage 3 of neuronal development [18]. There is an accumulating body of evidence suggesting that insulin-like growth factor 1 (IGF-1) is a critical inducer of axonal outgrowth at the nerve growth cone of cultured neurons [19]. In support of this, Sosa et al. (2006) demonstrated that the activation of the IGF-1 receptor (IGF-1R) is necessary for the establishment of neuronal polarity in cultured hippocampal neurons [20]. In addition to regulating neuronal polarity, IGF has also been found to have a proliferative and anti-apoptotic effect on the nervous system.

The polarizing effect of IGF-1 binding to the IGF-1 receptor appears to be mediated by the activation of phosphatidylinositol 3-kinase [18,20] as well as downstream effectors such as Cdc42 [20] and Akt (also called protein kinase B) [18]. These proteins in turn initiate a cascade of downstream signaling, eventually affecting microtubules and actin as well as their involvement in the direction of growth cone migration, thereby influencing cortical cell polarity [21].

Outside of the CNS, IGF-1 has been found to interact favorably with the widely distributed extracellular matrix (ECM) protein vitronectin during the migration of breast cancer cells as well as in epithelial cells in the cornea [22,23]. Together with IGF-binding proteins (IBFBP), IGF-1 and vitronectin form a multiprotein complex which acts to coactivate the IGF-1 receptor [23]. Vitronectin has also in itself been attributed an important role in spinal motor neuron differentiation [24] as well as in the differentiation of cerebellar granular cells [25]. However, whether an interaction between IGF/IGFBP/vitronectin is important for neuronal polarity, survival, or regeneration in the CNS remains unknown. Of note, vitronectin is clearly downregulated for four days following traumatic brain injury in rat [26]. Whether this downregulation has a negative impact on the potential positive effects of the posttraumatic IGF-1 upregulation after brain injury is not known. It is also unknown if modulating the levels and presence of vitronectin after brain injury could have a positive effect in brain injury.

In this study, we use a previously described in vitro neurotrauma model developed by our group [27]. In this model, single-layer cultured neurons can be traumatized in vitro and their posttraumatic reactions observed regarding cell degeneration/death and regeneration by new neurites post trauma. We use this system to test the hypothesis that the IGF-1/IGFBP-2/vitronectin system has a positive effect on neurite growth and polarization.

## 2. Materials and Methods

### 2.1. Primary Hippocampal Cell Culture

All animal studies are conducted in accordance with the guidelines of the regional ethics committee for animal research at the Karolinska Institutet, Stockholm, Sweden.

Sprague-Dawley rats (B&K Universal AB, Sollentuna, Sweden), kept under standard laboratory conditions, were sacrificed on the 18th gestational day using carbon dioxide, and the hippocampi were dissected from the fetuses (*n* = 30). Dentate gyrus, CA1, CA2, and CA3 regions were included in the dissected parts use for culture. The cultures were prepared as follows. The dissected hippocampi were incubated at 37 °C for 15 min in 0.1% trypsin (Invitrogen) diluted in Ca^2+^-Mg^2+^-free Hank’s Balanced Salt Solution (pH 7.3) and subsequently triturated through a narrowed Pasteur pipette. Cell suspensions were then seeded on thin cover slips in 35-mm tissue culture dishes (Corning, New York, NY, USA) at a cell density of 0.17 × 10^5^ cells/cm^2^. Prior to seeding, the cover slips were coated with 0.1 mg/mL poly-L-lysin hydrobromide (MW 3–7 × 10^4^; Sigma, Chemical Co., St. Louis, MO, USA) or vitronectin (Sigma-Aldrich, Stockholm, Sweden, 0.5 µg/cm^2^) and subsequently washed twice in distilled water. The cells were grown in 2 mL Neurobasal medium and were supplemented with B27, 1:50, (NB B27), 15 μg/ml gentamicin, and 2 mM L-glutamine (all from Invitrogen). The cultures were maintained in an incubator providing 5% CO_2_ at 37 °C. The growth medium was never changed and no re-feeding was done during the experimental period. In cultures treated with IGF-1 (GroPep, Adelaide, Australia) and IGFBP-2 (GroPep, Adelaide, Australia) these substances were added on the first day after seeding the hippocampal neurons, IGF-1 at a concentration of 10 ng/mL resolved in DMEM + 0.01% BSA and IGFBP-2 at a concentration of 100 ng/mL resolved in DMEM + 0.01% BSA.

### 2.2. In Vitro Trauma

A subset of cell cultures was subjected to in vitro trauma. The method employed was previously described in detail [27]. Briefly, the cell monolayer of hippocampal neurons was exposed to a shock wave cavitation trauma (SWCT). A pulsed Nd-YAG laser (wavelength 1064 nm) produced laser bursts with energy measuring 500–600 mJ. The laser was directed toward a copper-coated fused silica window. When subjected to the laser, the inner layer of the copper vaporized. The expansion of copper vapors accelerated a piece of the superficial layer of the metal—the flyer-plate. One well at a time was placed on top of the copper-silica window, covered by a projector transparency. The well bottom was hit by the accelerating flyer-plate and a resulting cavitation developed at the bottom and the surface of the medium, in turn inducing trauma in the cell monolayer. An earlier study showed that top cavitation always leads to droplet-formation on the lid above the well [28]. This top cavitation droplet was used as an inclusion criterion for continued observation of the cell culture in question. Without a droplet, the cell culture was not further studied. The model was described in detail in earlier studies [27,28,29].

### 2.3. Immunohistochemistry

At 1, 2, 3, 4, 6, and 7 days after treatment with ± IGF-1/IGFBP-2 on n-L-lysin or vitronectin and subjection to in vitro trauma or not, the culture (*n* = 6 culture wells per group and time point) were fixed in 4% formalin for 1 min and rinsed in 0.01 M PBS for 10 min. Thereafter, they were incubated in a humid chamber at 4 °C for 24 h with rabbit polyclonal antibody against MAP2 (Millipore, dilution 1:1000) and mouse monoclonal antibody against beta III (anti-beta III tubulin antibody neuronal marker, Abcam, dilution 1:2000). The cell culture wells were then rinsed in 0.01 M PBS and incubated for 60 min at 20 °C with 0.01% PBS + 0.1% sodium azide + 0.3%Triton containing Cy3-conjugated donkey anti-rabbit IgG (Jackson ImmunoResearch, inc.PA, USA; dilution 1:500) and Cy2-conjugated donkey anti-mouse IgG (Jackson ImmunoResearch, inc.PA, USA; dilution 1:200). The coverslips were gently removed from the wells and mounted on slides with mowiol 4-88 (Polysciences). Coverslips from 12 wells for each treatment group (poly-L-lysin or vitronectin ± IGF-1/IGFBP-2 treatment ± in vitro trauma) were handled in this way and used for microscopic analysis.

In cultures used for measurements of polarization, the same procedure was conducted at a 6 h, 12 h, 18 h, 24 h, and 48 h after treatment with ± IGF-1/IGFBP-2 and poly-L-lysin or vitronectin. Coverslips from 6 wells for each treatment group (poly-L-lysin or vitronectin ± IGF-1/IGFBP-2 treatment) were handled in this way and used for microscopic analysis. A total of 50 cells per well was measured regarding polarization.

### 2.4. Microscopy Analysis

**Figure 1:**
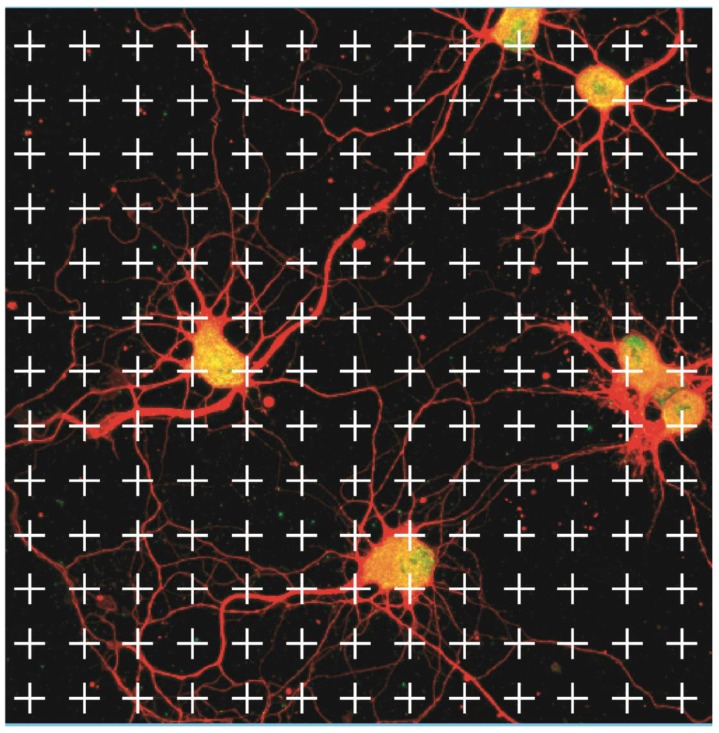
Example of a grid used for measurements of neurite density in primary hippocampal neuron cultures. A cross covering one or more neurites was considered one observation. A cross placed above a cell nucleus was not counted. To exemplify, arrows mark crosses that should be considered one observation, while arrowheads marks crosses that should be considered no observation. (red = beta III tubulin, green/yellow = MAP2).

Analysis was carried out with a Nikon D-eclipse C1 confocal microscope. For measurements of neurite density in non-traumatized cultures, six to eight randomly selected areas with healthy looking neurons were chosen from each coverslip on which laser scanning was performed, and microphotographs saved as JPEG files. A standardized grid was superimposed on each single JPEG (Figure 1). If one or more neurites passed over one cross, this was considered one observation. A cross placed over a cell body was not considered an observation. In this way a measurement of neurite density was achieved. The analysis of the grid with superimposed JPEGs was performed by a blinded observer. A mean value from all observations in each coverslip was calculated and handled as one value in the statistical analysis/graphical presentations.

**Figure 2:**
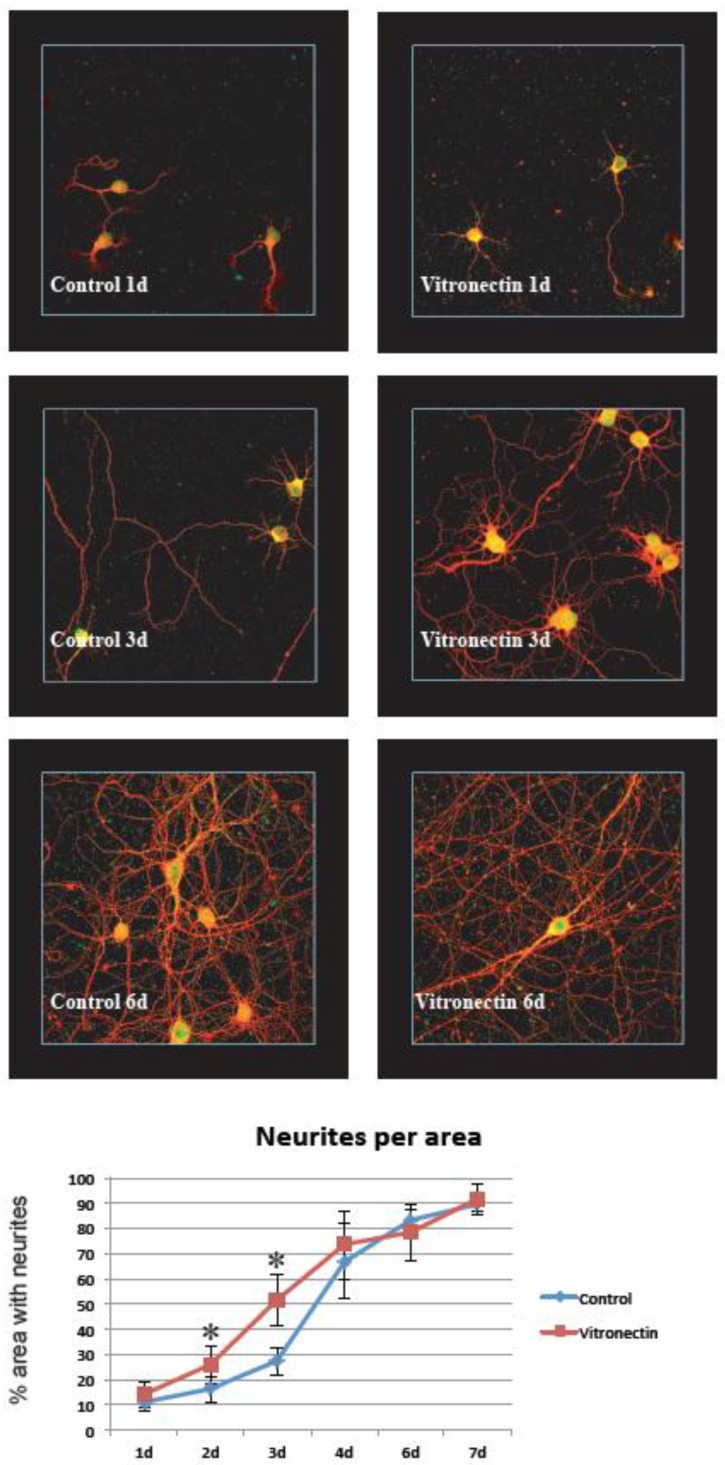
Hippocampal neurons grown on the vitronectin substrate showed an early increase in neurite growth compared with the controls. At 2 and 3 days there was significantly (*) more neurites per area (calculation of neurite density explained in Figure 1 and in the methods section) when neurons were grown on vitronectin compared to the controls (*n* = 6 (6 wells per time point and treatment), two-way ANOVA, Bonferroni post hoc test, *p* < 0.05) (red = beta III tubulin, green/yellow = MAP2).

**Figure 3:**
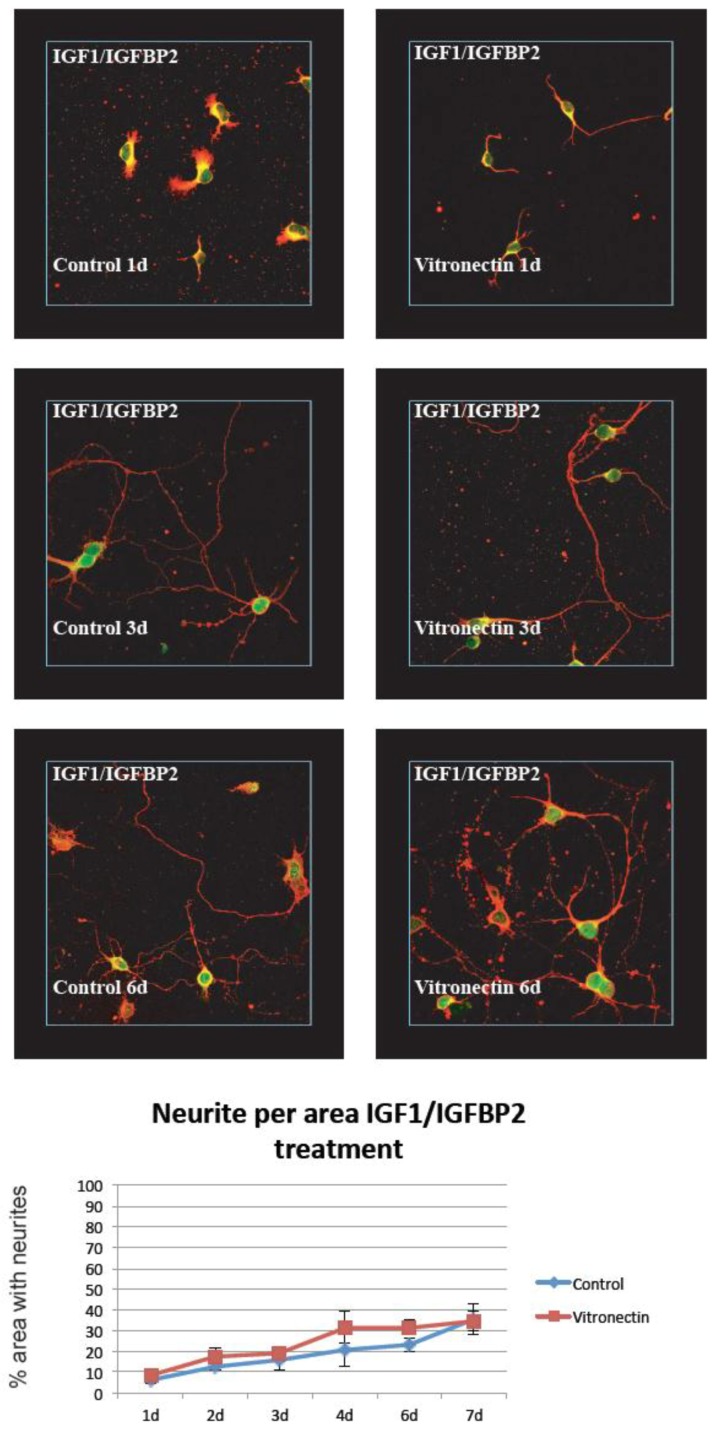
The addition of IGF-1/IGFBP-2 to cultured hippocampal neurons did not show any increase in neurite numbers per area, but rather showed a decrease compared with the controls and in total and a marked decrease in neurites per area compared with the hippocampal neurons grown on standard coating or vitronectin (*n* = 6 (6 wells per time point and treatment), two-way ANOVA with Bonferroni post hoc test, *p* < 0.05) (red = beta III Tubulin, green/yellow = MAP2).

**Figure 4:**
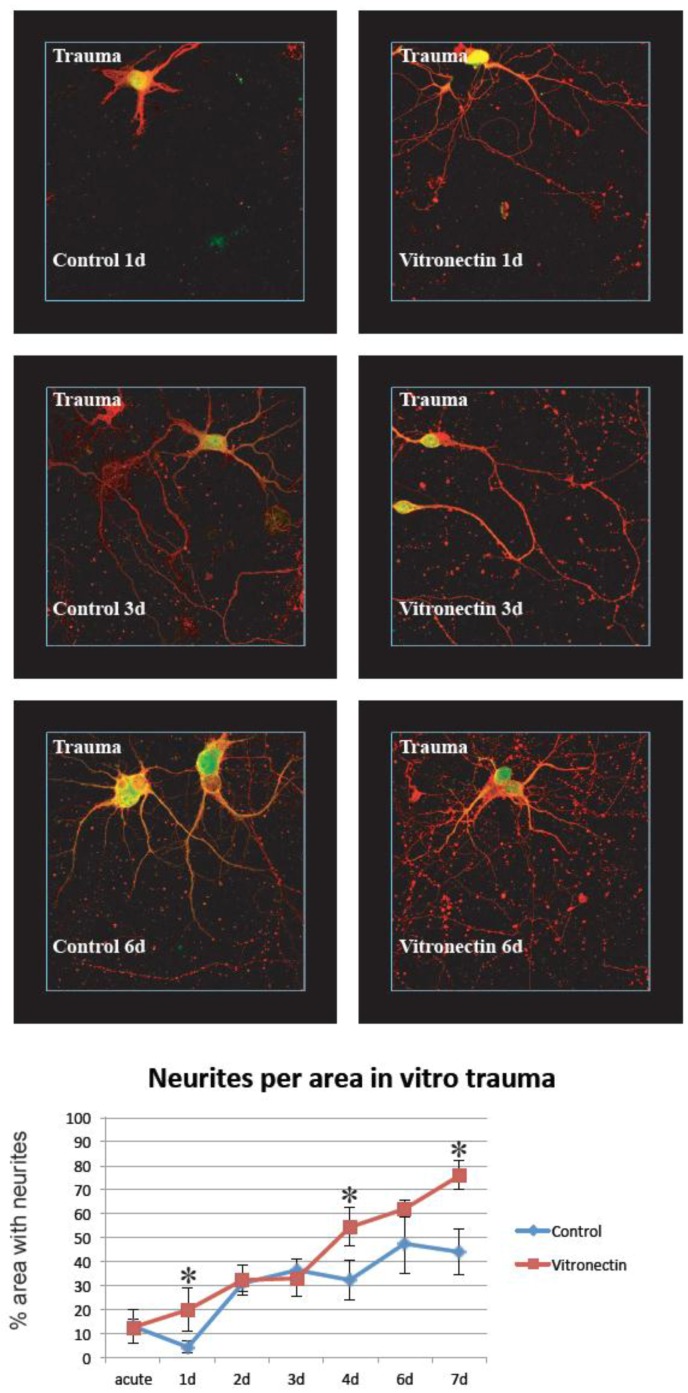
After in vitro trauma to cultured hippocampal neurons, neurite extension was calculated at the border between viable cells and the central area of injury. Neurons regrew neurites slightly better on vitronectin than on the standard coating. At 1, 4, and 7 days after trauma, there were significantly (*) more neurites when neurons were grown on vitronectin compared to the control coating (*n* = 6 (6 wells per time point and treatment), two-way ANOVA with Bonferroni post hoc test, *p* < 0.05) (red = beta III tubulin, green/yellow = MAP2).

**Figure 5:**
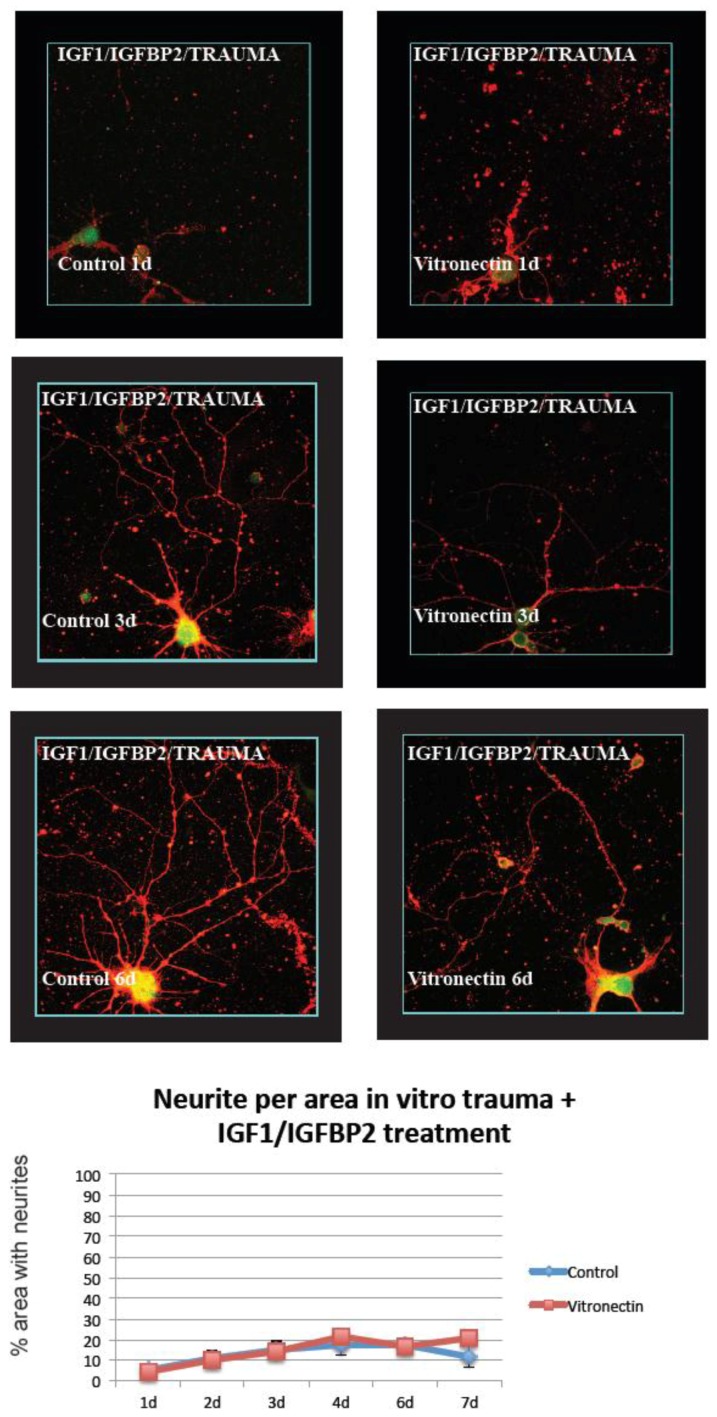
After in vitro trauma and treatment with IGF-1/IGFBP-2, hippocampal neurons neurite extension was calculated at the border between viable cells and the central area of injury. No difference in neurite extensions was shown on vitronectin compared to the standard coating, and a decrease in neurite density was shown compared to neurons cultured without IGF-1/IGFBP-2 treatment (*n* = 6 (6 wells per time point and treatment), two-way ANOVA with Bonferroni post hoc test, *p* < 0.05) (red = beta III tubulin, green/yellow = MAP2).

In the traumatized cultures, the same principle was used but here the area at the border between the injured zone and the non-injured zone was chosen. Thus, the regenerating neurites from neurons subjected to in vitro trauma were analyzed (results are shown in Figure 2, Figure 3, Figure 4 and Figure 5)

**Figure 6:**
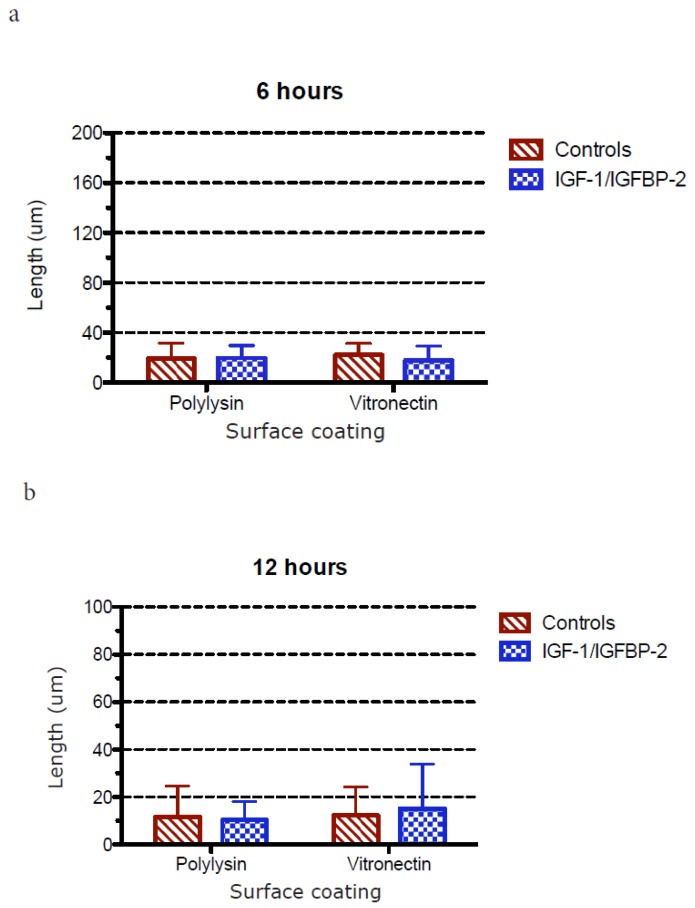

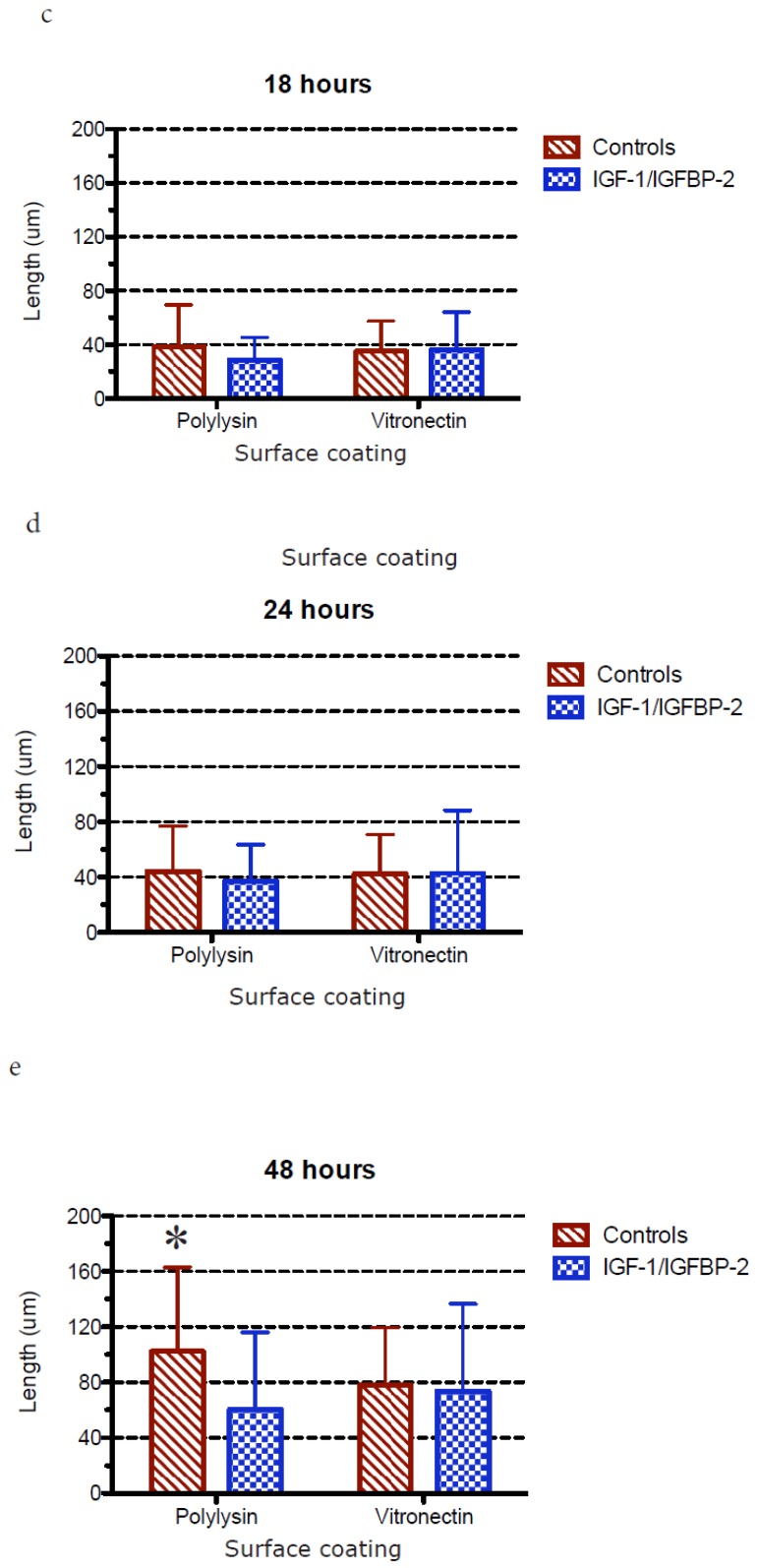
(**a**)–(**e**) The calculation of the longest neurite at different time points after injury. The length of the neurites was calculated as described in the methods section. Six wells for each treatment and time point were used for measurements and five cells per well were measured. Overall, no major differences were observed between growth on vitronectin and poly-L-lysin with or without treatment with IGF-1/IGFBP-2, except at later time points when growth on poly-L-lysin was found to be favorable both compared to vitronectin coating and to treatment with IGF-1/IGFBP-2. This is because, at 48 h, growth on poly-L-lysin showed statistically significant (*) longer neurites compared to growth on vitronectin both with and without IGF-1/IGFBP-2 treatment (one-way ANOVA with Bonferroni post hoc test, *p* < 0.05).

The polarization of hippocampal neurons grown on poly-L-lysin or vitronectin and treated with or without IGF-1/IGFBP-2 treatment was studied in two different ways:

1: The length of the longest neurite was measured. The first 50 healthy-looking neurons encountered at each coverslip, without selecting for longer neurites, were selected. Neurons in larger groups whose neurites could not be accurately distinguished from each other were excluded, as were neurons with neurites so long that they could not fit into a single microphotograph. Thereafter, the longest neurite was measured. A mean value from all of the longest measured neurites from each coverslip was calculated and treated as one value in the statistical analysis/graphical presentations (results are shown in Figure 6).

**Figure 7:**
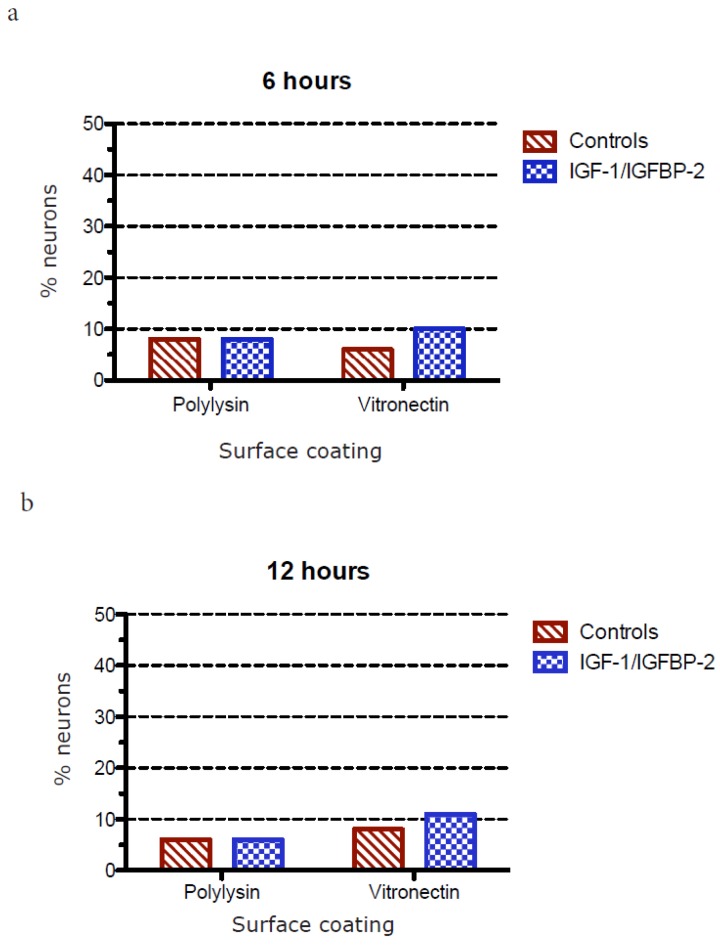

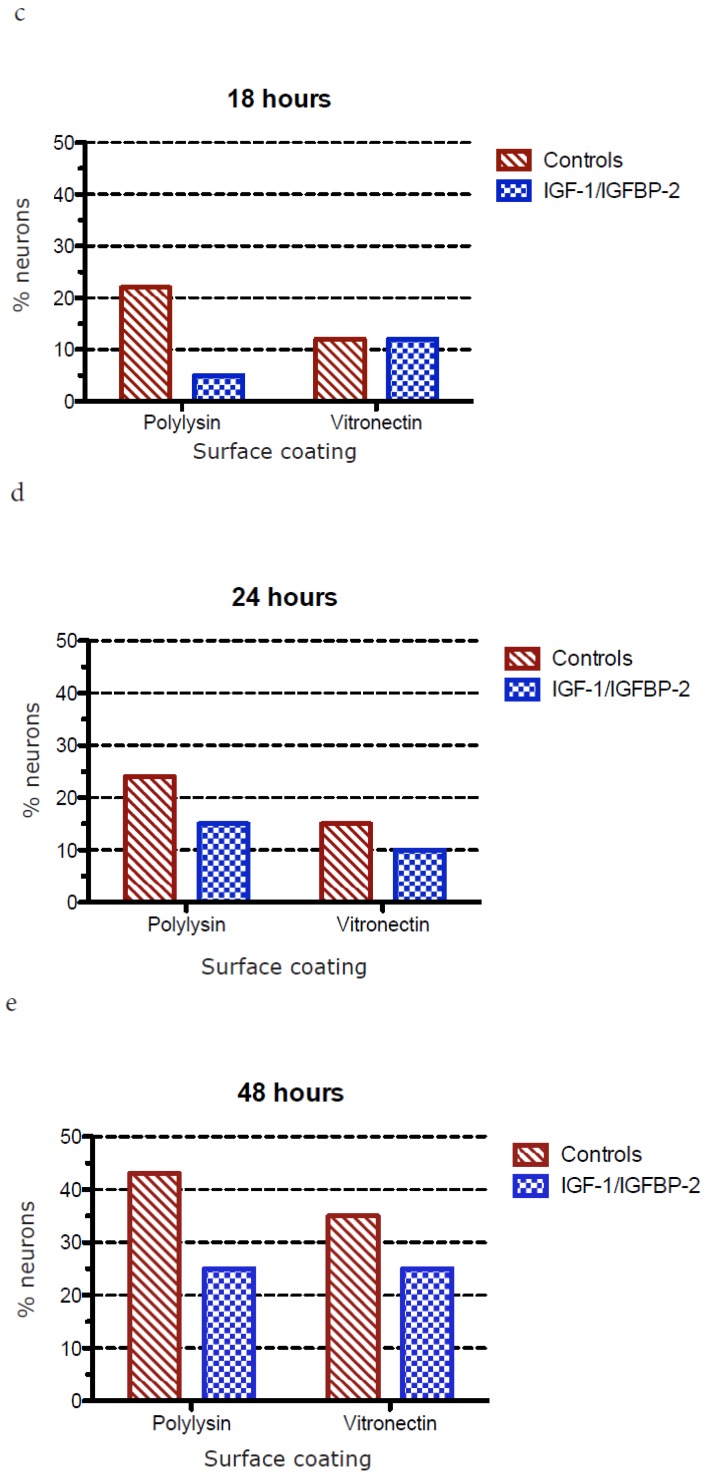
(**a**)–(**e**) The proportion in percentage of neurons at differentiation stage 3 or higher grown on poly-L-lysin or vitronectin and treated with or without IGF-1/IGFBP-2. Six wells for each treatment and time point were used for measurements and five cells per well were measured. At later time points, there was a trend towards growth on poly-L-lysin without IGF-1/IGFBP-2 treatment being favorable to reaching differentiation stage 3 or higher.

**Figure 8:**
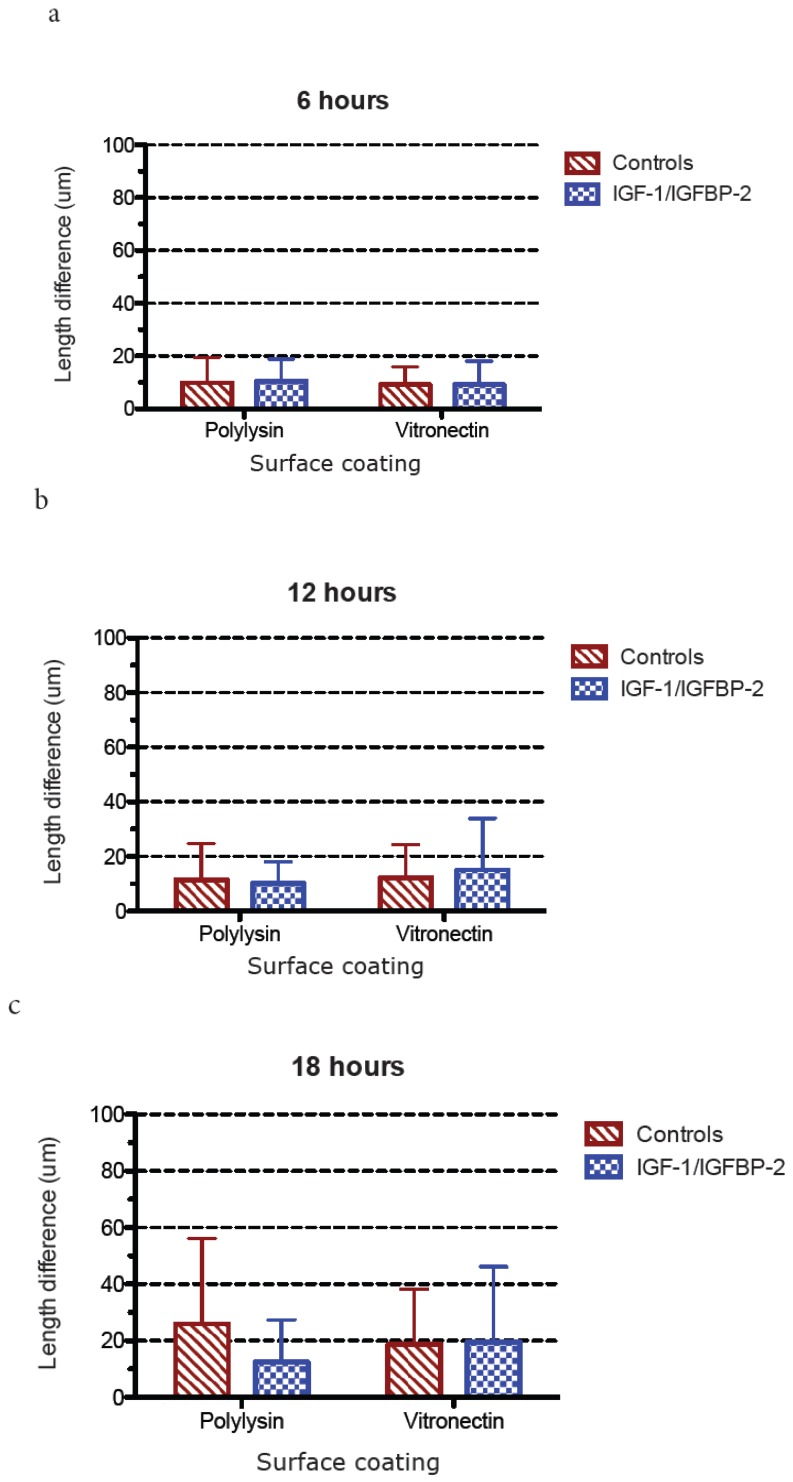

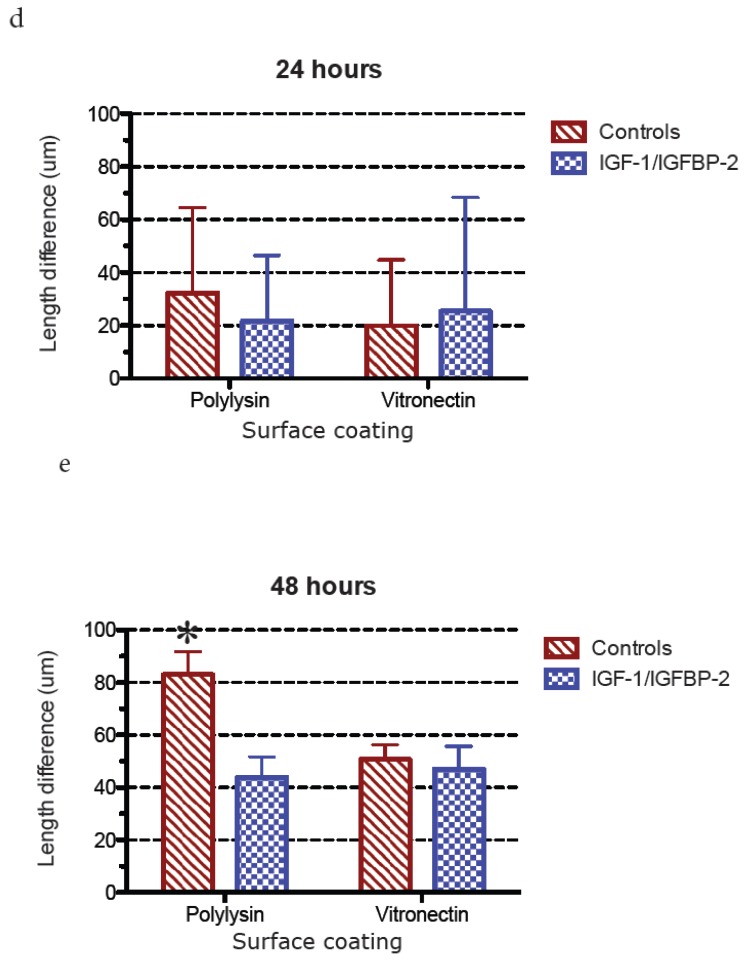
(**a**)–(**e**) The mean and standard deviation of the difference in length in micrometers between the longest neurite and the average length of three minor neurites are displayed. Six wells for each treatment and time point were used for measurements and five cells per well were measured. At later time points, there is trend towards poly-L-lysin without IGF-1/IGFBP-2 treatment being favorable for polarization. Also, at 48 h, growth on poly-L-lysin showed statistically significant differences (*) in micrometers between the longest and minor neurites compared to growth on vitronectin both with and without IGF-1/IGFBP-2 treatment (one-way ANOVA with Bonferroni post hoc test, *p* < 0.05).

2: Differentiation stage 3 was defined as when the longest neurite exceeded the length of the average minor neurite by 20 μm [20]. This was calculated as the difference in length between the longest neurite and the average length of up to three minor neurites of the same neuron. A mean value from all measurements from each coverslip was calculated and treated as one value in the statistical analysis/graphical presentations (results are shown in Figure 7 and Figure 8).

### 2.5. Statistical Analysis

For statistical analysis, GraphPad Prism 7.0 software was used (GraphPad Software, La Jolla, CA, USA). Two-way ANOVA (Figure 2, Figure 3, Figure 4 and Figure 5) and one-way ANOVA (Figure 6, Figure 7 and Figure 8) analyses were used with the addition of the Bonferroni post hoc test for calculations of significant differences between groups. A *p*-value < 0.05 was considered significant.

## 3. Results

### 3.1. Neurite Growth on Poly-L-lysin Compared to Vitronectin

It was easy to establish clean, primary hippocampal neuron cultures showing the stable growth of neurites on the control poly-L-lysin coating. When compared to growth on the vitronectin coating, we found that this substrate increased the establishment of neurites early after the seeding of the cells, and the density of neurites per area differed significantly at 2 and 3 days after the start of treatment (*p* < 0.05). However, at later time points there was no difference between the groups and the cultures both reached around 90% neurite density after 7 days of growth (Figure 2). This was in sharp contrast to growth on poly-L-lysin and vitronectin and after treatment with IGF-1/IGFBP-2, where the neurite density never reached higher than about 30% of the area and where no significant difference between growth on vitronectin or poly-L-lysin was observed (Figure 3).

### 3.2. Neurite Growth after In Vitro Trauma

When cultures were subjected to in vitro trauma, vitronectin again was found to be a favorable growth substrate compared to poly-L-lysin, with significant differences between the two groups especially at later time points after injury. Compared to non-injured cultures, the maximum neurite density was delayed and reached lower total levels, around 75% density for neurons grown on vitronectin, at the end of the experiment (Figure 4). Again, treatment with IGF-1/IGFBP-2 showed a dramatically less dense growth of neurites per area compared to growth on vitronectin or poly-L-lysin, only after trauma, and reached only around 20% density with no differences between the groups (Figure 5).

### 3.3. Measurements of Neurite Polarization

In an attempt to investigate whether the less dense neurite network after IGF-1/IGFBP-2 treatment was due to a higher degree of polarization, e.g., the formation of a more mature axon-like neurite instead of a network of neurites, we investigated the potential polarization of neurites in two different ways after treatment with IGF-1/IGFBP-2 on poly-L-lysin and vitronectin, firstly by measuring the length of the longest neurite and secondly by measuring when the longest neurites exceeded the length of the average minor neurite by 20 μm, which has been defined as differentiation stage 3 in the polarization of neurons [20].

Figure 6 shows the measurements of the longest neurite in cultures with poly-L-lysin or vitronectin coating and ± IGF-1/IGFBP-2 treatment. No major differences could be observed between growth on the different kinds of coating or with or without treatment with IGF-1/IGFBP-2, with the exception of later time points when growth on poly-L-lysin showed a favorable trend compared with growth on vitronectin and compared to treatment with IGF-1/IGFBP-2. This is because, at 48 h, growth on poly-L-lysin showed statistically significant longer neurites compared to growth on vitronectin both with and without IGF-1/IGFBP-2 treatment (two-way ANOVA, *p* < 0.05).

When studying the proportion in percentage of neurons at differentiation stage 3 or higher, a trend towards growth on poly-L-lysin without IGF-1/IGFBP-2 treatment being favorable compared with treatment and growth on vitronectin was observed at later time points (Figure 7). Also, when measuring polarization, as the difference in length in micrometers between the longest neurite and the average length of three minor neurites, the trend was consistent: at later time points, growth on poly-L-lysin without IGF-1/IGFBP-2 treatment was favorable in terms of the polarization of neurons, with a statistically significant difference in favor of poly-L-lysin without IGF-1/IGFBP-2 treatment at 48 h (two-way ANOVA, *p* < 0.05) (Figure 8).

## 4. Discussion

In this article, we investigated the potential positive impact of the IGF-1/IGFBP-2 system on neurite growth in vitro under normal conditions and after in vitro trauma. We also investigated the potential role of vitronectin as a supportive factor for the neurite growth-stimulating capacity of IGF-1/IGFBP-2. Vitronectin as a growth substrate was shown to be slightly favorable for neurite growth compared to the control substrate both without and with in vitro trauma, but IGF-1/IGFBP-2 had only a marginal additive effect, both under the control conditions or after trauma. Since neurite growth was measured as the number of neurites per area, a less dense neurite network could potentially be attributed to a higher degree of polarization of the neurons after treatment with IGF-1/IGFBP-2. This hypothesis was also tested, but a higher degree of polarization was not observed in IGF-12/IGFBP-2-treated neurons grown on vitronectin. Rather, the opposite was found, with a higher degree of polarization after growth on the control substrate poly-L-lysin at a few of the observed time points.

In our study, both IGF-1 and IGFBP-2 were added to investigate the potential role of IGF-1 and vitronectin in neuronal survival and regeneration. The presence of both IGF and IGFBPs together with extracellular matrix molecules, of which vitronectin is one strong candidate [30,31,32], has without doubt been demonstrated to be beneficial for cell survival and growth [33,30]. In the CNS, the potential role for this interaction is less well documented. Both IGF and IGFBPs are present after TBI [1,2,4] and the differentiation of cerebellar granular cells [25] and motor neurons are dependent on vitronectin [24]. Our findings of vitronectin as favorable compared to the control (Figure 2 and Figure 4) was thus not unexpected. Recent studies have also shown that intraventricular administration of IGF-1 after experimental traumatic brain injury results in the enhanced generation of immature hippocampal neurons in the granular layer of the dentate gyrus [11] and that an impaired IGF-1 system contributes to neurodegeneration in inflammatory disorders in CNS [34]. These findings could support the notion that IGF-1 is of importance in CNS injuries, even though the picture is far from fully understood regarding the fine tuning of the IGF/IGFBP/vitronectin complex in the nervous system. 

The role of IGFBP in our study is of interest since IGFBPs can either inhibit or stimulate the actions of IGF-1, depending, e.g., on cell type [35,36,37]. One possible important factor behind the distinct downregulation of neurite growth after the addition of IGF-1/IGFBP-2 in our study could thus be the interaction between IGF-1 and IGFBP-2. On one hand, has it been shown that the binding affinity of IGFs to IGFBPs decreases when IGFBPs are bound to ECM [38] and possibly also to vitronectin since cancer cell migration and survival are enhanced by the IGF/IGFBP/vitronectin complex [32], which would potentially be beneficial for nerve growth and survival in our study. On the other hand, it is clearly shown that IGFBPs have a high affinity for IGFs and as such can function as decoys for free IGF, thereby reducing the levels of free IGF (see Allard and Duan for a review on IGFBPs [39]). In the neuroscience field, one study pointing in this direction came from Wilczak et al. (2003), whose findings showed that the levels of free IGF-1 in the ventral horn of the spinal cord was lower in ALS patients than in healthy controls, and that this reduction of free IGF-1 coincided with corresponding high levels of IGFBPs in ALS patients compared to a control (64% higher levels of IGFBP-2 in ALS compared with controls), which the authors linked to higher motor neuron death in ALS [17]. Thus, the marked decrease in neurite growth shown by us after treatment of hippocampal neurons with IGF-1/IGFBP-2 could in fact be a result of IGFBP-2 restricting IGF-1 to exert its growth-promoting role on neurons.

## 5. Conclusions

We conclude that vitronectin has a supportive effect on neurite growth compared with the controls both under normal culture conditions and after in vitro trauma. We also conclude that IGF-1/IGFBP-2 treatment had no additive effect to this growth.
